# Remedy for Ferociousness in a Dog

**Published:** 1851-01

**Authors:** 


*Remedy for Ferociousness in a Dog.*—In one of the Cincinnati papers, we find an account of an attack of a ferocious dog upon a little child. "The dog seized the child by the throat," we are told, "and the more he was pounded so make him let go, the harder he held on. The people broke the dog's back, and after inserting a lever into his mouth, pried his !aws open and released the sufferer, but not till her throat was mangled." There is a *sure* remedy in such cases, which should be known by every one. We hear of the case often, and it would seem that persons at these times are very apt to forget the disposition of the animal. Now, if instead of pulling upon the dog, to disengage him when his jaws are set upon anything, a sponge or cloth, wet with *strong spirits of hartshorn,* be applied to his nostrils, he will instantly relax his hold.—*Boston Med. and Sur. Jour.*

